# The specificity of homomeric clustering of CD81 is mediated by its δ‐loop

**DOI:** 10.1002/2211-5463.12187

**Published:** 2017-01-19

**Authors:** Yahya Homsi, Thorsten Lang

**Affiliations:** ^1^ Membrane BiochemistryLife & Medical Sciences (LIMES) InstituteUniversity of BonnGermany

**Keywords:** CD151, CD9, membrane protein clusters, tetraspanin web, tetraspanin‐enriched microdomains, tetraspanins

## Abstract

Tetraspanins are cell membrane‐scaffolding proteins interacting with one another and a repertoire of interaction partners. Through these interactions, they form extended molecular networks as tetraspanin webs or tetraspanin‐enriched microdomains. Microscopic data suggest that these networks contain tetraspanin clusters, with poor overlap between clusters formed by different tetraspanins. Here, we investigate the possibility of targeting tetraspanins CD9 or CD151 to clusters formed by the tetraspanin CD81. We find that the δ‐loop from the large extracellular domain of CD81 is sufficient for targeting of CD9/CD151 to CD81 clusters. Moreover, in a pull‐down assay, CD9 coprecipitates more CD81 when it carries the CD81 δ‐loop. In conclusion, the information for forming homomeric CD81 clusters is encoded in the δ‐loop.

**Abbreviations**
LEL: large extracellular loop
PCC: Pearson correlation coefficient
PDB: protein data bank
ROIs: regions of interest
SEL: small extracellular loop
TEMs: tetraspanin‐enriched microdomains
TMA‐DPH: 1‐(4‐tri‐methyl‐ammonium‐phenyl)‐6‐phenyl‐1,3,5‐hexatriene‐p‐toluenesulfonate
VD: variable domain
VMD: virtual molecular dynamics

Tetraspanins are a family of small membrane proteins expressed in animals, plants and fungi, with 33 members in humans. They act as scaffolding proteins in the cell membrane, forming large interaction networks with one another and other molecules. Tetraspanin interactions partners are diverse, including integrins/other adhesion molecules, members of the immunoglobulin superfamily, signalling receptors and gangliosides 1, 2. This wide repertoire of factors explains why tetraspanins play so many roles in physiological as well as in pathophysiological processes, as cell proliferation, signal‐transduction, vesicle trafficking, cell–cell fusion, adhesion, spreading, migration, cancer, infectious diseases and host–pathogen interactions 3, 4, 5, 6, 7. They are also considered to be ‘master organizers’ of the plasma membrane.

Commonly, tetraspanin interactions are identified and classified into primary and secondary interactions employing immunoprecipitation with detergents of variable strength. Robust and direct interactions formed between a tetraspanin and a specific partner are primary complexes, while interactions only stable under milder solubilization conditions are secondary, as the many tetraspanin–tetraspanin associations that crosslink primary complexes to large tetraspanin webs 8. Secondary interactions are further stabilized by a third level of weak and indirect interactions 6. These are based on hydrophobic palmitates attached through palmitoylation to tetraspanin residues close to the inner plasmalemmal leaflet 9, 10.

Physical dimensions of TEMs studied by electron microscopy reveal that CD63‐ and CD9‐enriched TEMs occupy an area of 0.2 μm^2^, although size and shape vary considerably 11. The majority of CD63 and CD9 form distal clusters. Super‐resolution light microscopy, focussing on the tetraspanins CD37, CD53, CD81 and CD82, determined a tetraspanin cluster size in the range of 100–150 nm 12, 13. As CD63‐/CD9‐clusters in electron microscopy, also in light microscopy individual members of the tetraspanin family form clusters largely devoid from other tetraspanins 13. Tetraspanin cluster segregation in microscopy likely reflects evidence from biochemical cross‐linking showing a higher level of tetraspanin homo‐dimers/higher homo‐oligomers when compared to hetero‐dimers 14.

There are also parallels between biochemistry and microscopy regarding the interaction strength between two partners. For instance, CD81 and its primary interaction partner EWI‐2 overlap stronger with each other than CD81 with its secondary interaction partner CD9 15. Moreover, CD53 and CD81 are in closer proximity to their primary interaction partners than to other tetraspanins 13.

Tetraspanins share a common structure composed of four transmembrane domains, small intracellular segments and two extracellular loops. The structure of the small extracellular loop is unknown, but the large extracellular loop (LEL) can be subdivided into five helical segments [16,17]. The structure and the conformational flexibility of the LEL of CD81 have been studied 17, 18, 19. Based on the crystal structure of the CD81 large extracellular loop it was proposed that CD81 dimerizes via a conserved hydrophobic interface 17. However, due to the antiparallel orientation of the two dimerizing proteins this mechanism can only account for dimers forming on opposed or extremely curved membranes. Deleting subdomains within the LEL it was shown that a small segment comprising the δ‐loop is required for directing CD81 molecules into CD81 clusters, possibly involving a CD81 dimerization step 15. However, it is not clear whether the δ‐loop is required for cluster stabilization or mediates the specificity in CD81 oligomerization.

Here, we report that the δ‐loop determines the specificity in clustering showing that the tetraspanins CD9 or CD151 are directed into CD81 clusters when they carry the CD81 δ‐loop.

## Materials and methods

### Constructs

Plasmids encoding for CD81‐GFP and CD9‐RFP were previously described (15; GFP is a monomeric variant from EGFP; RFP is a monomeric variant carrying at its C terminus a myc‐tag). CD9_Chim_‐RFP is based on CD9‐RFP and was generated by fusion PCR, substituting the CD9 δ‐loop located between cysteine residues 167 and 181 with the CD81 δ‐loop region (between cysteine residues 175–190). For the CD151‐RFP construct, the CD151 sequence was amplified from a plasmid described in Ref. 20, C‐terminally fused to RFP (see above), and cloned into the pEGFP_C1 vector (6084‐1; Clonetech, Mountain View, CA, USA). CD151_Chim_‐RFP was generated from CD151‐RFP via fusion PCR, by substituting the CD151 δ‐loop between the cysteine residues 192 and 208 with the CD81 δ‐loop (see above). All constructs were verified by sequencing, using as reference the respective homo sapiens sequences (CD81, NP_004347.1; CD9, NP_001760.1; CD151, NP_001034579.1).

### Cell culture and membrane sheets

Jurkat E6.1 and HepG2 cells were maintained and transfected as described previously 15. For each construct, 30 μg of plasmids was used for transfections. From transfected Jurkat E6.1 or HepG2 cells membrane sheets were generated in ice‐cold sonication buffer after 1 day or 2 days, respectively, as previously described 15.

### Antibodies

In western blot experiments, mouse monoclonal anti‐CD81 (1.3.3.22) antibody (sc‐7637; Santa Cruz, Dallas, TX, USA) and rabbit polyclonal anti‐RFP antibody (600‐401‐379; Rockland, Limerik, PA, USA) were used for detecting CD81 and RFP fusion proteins, respectively, in combination with the secondary antibodies goat‐anti‐mouse IgG‐HRP (sc‐2031; Santa Cruz) and goat‐anti‐rabbit IgG‐HRP (sc‐2030; Santa Cruz).

### Microscopy

Confocal scanning microscopy was done on an Olympus FluorView 1000 microscope (Olympus, Tokyo, Japan) used previously 15. In brief, intact cells coexpressing CD81‐GFP in combination with CD9‐RFP or CD9_Chim_‐RFP were adhered on poly‐L‐lysine‐coated glass coverslips as described previously 15. Cells were directly fixed for 30 min at RT with 4% paraformaldehyde (PFA) in PBS (137 mm NaCl, 2.7 mm KCl, 8.1 mm Na_2_HPO_4_, pH 7.4), treated with NH_4_Cl in PBS for quenching of PFA, and imaged in PBS. For imaging, the glass‐adhered plasma membrane was positioned into the focal plane. GFP‐, RFP‐ and DIC images were recorded with a pixel size of 103 nm, scanning a 300 pixel × 300 pixel area. GFP and RFP were excited by 488 nm and 543 nm lasers, respectively.

Epifluorescence microscopy was performed using the same microscopic equipment and settings described previously 15. In brief, membrane sheets from double‐transfected cells were generated by a 100‐ms sonication pulse, fixed with PFA and treated with 50 mm NH_4_Cl in PBS for quenching of PFA. Imaging was performed in PBS containing TMA‐DPH [1‐(4‐tri‐methyl‐ammonium‐phenyl)‐6‐phenyl‐1,3,5‐hexatriene‐p‐toluenesulfonate (T204; Thermofisher, Waltham, MA, USA)] for visualizing the membranes. In addition we added tetraspeck beads (Invitrogen, Carlsbad, CA, USA) enabling to correct for lateral shifts. Images were recorded in the green (CD81‐GFP), red (CD9‐RFP, CD9_Chim_‐RFP, CD151‐RFP or CD151_Chim_‐RFP) and blue (TMA‐DPH) channels. In the green channel, exposure times for Jurkat T and HepG2 membrane sheets were 1s and 100 ms, in the red channel exposure times were 2s and 1s, respectively.

The similarity between the signal distribution in the green and the red channels was quantified by calculation of the Pearson correlation coefficient (PCC) as previously described 15. In brief, to avoid any bias in analysis, the regions of interest (ROIs) were positioned in the TMA‐DPH or DIC image that illustrate the membrane or cellular shape (without knowing the distribution of the green and the red signals), respectively. After transferring the ROIs to the green and red channels, it was checked whether any highly fluorescent structures as bright artefacts, tetraspeck beads, organelles or membrane remnants are located in the ROIs. If so, the ROI was slightly moved to a different location or if this was not possible, the membrane sheet or cell was excluded from the analysis. Images are shown at arbitrary scalings.

### Immunoprecipitation

10^7^ Jurkat E6.1 cells were transiently mock transfected or transfected with Lifeact‐RFP (Lifeact 21 C‐terminally fused to mRFP and inserted between *Age*I/*Not*I restriction sites in the vector mRuby‐N1; 54581 Addgene), CD9‐RFP or CD9_Chim_‐RFP. After 2 days, cells were harvested, washed once with 5 mL HEPES buffer (25 mm HEPES, 150 mm NaCl, 5 mm MgCl_2_, pH 7.2) and lysed in 1 mL HEPES buffer supplemented with 1% CHAPS (C5070; Sigma‐Aldrich, St. Louis, MO, USA), 10 μm PMSF and protease inhibitor cocktail (Roche, Mannheim, Germany). The cell lysates were rotated at 4 °C for 30 min and then centrifuged for 5 min at 3800 ***g***. The supernatants containing the solubilized proteins were used for immunoprecipitation realized by adding 25 μL RFP‐Trap^®^_A agarose beads (rta‐10; ChromoTek, Planegg‐Martinsried, Germany). The solutions were incubated under rotation at 4 °C for 1 h. Afterwards, beads were collected by a quick centrifugation step (2 min at 2500 ***g*** at 4 °C) and washed twice with HEPES buffer supplemented with protease inhibitor cocktail and 10 μm PMSF. The pulled down proteins were then subjected to western blot analysis under nonreducing conditions. We subsequently immunoblotted for CD81 and RFP‐labelled constructs using mouse monoclonal anti‐CD81 (1.3.3.22) antibody overnight at 4 °C and rabbit polyclonal anti‐RFP antibody for 1 h at RT. Detection of the respective primary antibodies was performed using HRP‐coupled secondary antibodies that were visualized on autoradiography films using a Luminol chemiluminescence kit (sc‐2048; Santa Cruz). Bands were quantified on scanned radioactive films. For quantification of the coimmunoprecipitated CD81, background values obtained from the Lifeact‐RFP control were subtracted. Western blot parts from the same membrane are shown at the same scaling.

## Results and Discussion

We previously set‐up an assay for analysing the microscopic overlap of tetraspanin constructs in the cell membrane of Jurkat T cells 15. In brief, Jurkat T cells coexpressing GFP‐ and RFP‐labelled tetraspanins were adhered to a glass‐surface and then exposed to a brief ultrasound pulse, which removes the upper cellular part, leaving behind the intact glass‐adhered native membrane. In this preparation, the mobility behaviours of membrane proteins has been shown to be unchanged when compared to intact cells 22, 23. As the preparation is two‐dimensional, imaging can be performed by simple epi‐fluorescence microscopy with a CCD camera at high signal‐to‐noise ratio, thus avoiding confocal microscopy‐based optical sectioning techniques (in combination with a photomultiplier) with a reduced signal‐to‐noise ratio. This becomes important when less potent fluorophores as RFP are recorded.

Quantifying the overlap between the pairs CD81‐RFP/CD81‐GFP and CD9‐RFP/CD81‐GFP yielded Pearson correlation coefficient (PCC) values of 0.6 and 0.35, respectively 15. Due to instrument noise and other factors, even double‐tagged proteins do not yield a PCC even close to ‘one’ (indicating identical images), but, for example, 0.63 24. On the other hand it should be considered, that due to the meso‐scale organization of protein clusters in multiprotein assemblies 25 also unrelated proteins show a PCC higher than ‘zero’, typically in the range below 0.1. Within the theoretical dynamic range between ≈ 0.1 and ≈ 0.6, the above PCC values for CD81‐RFP/CD81‐GFP and CD9‐RFP/CD81‐GFP suggest that the overlap between these constructs is perfect and weak, respectively.

**Figure 1:**
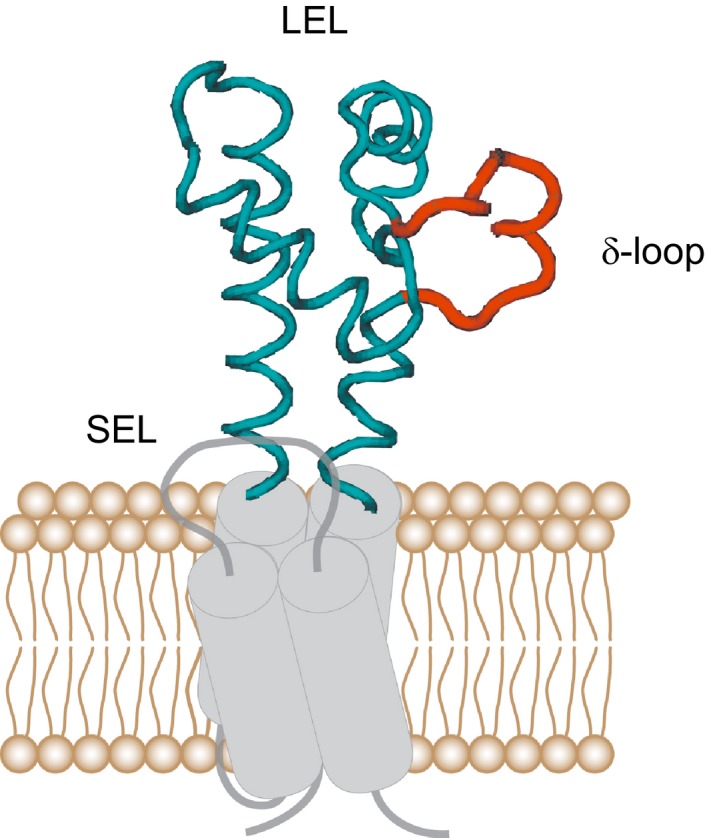
Tetraspanin domain structure illustrated using as example the LEL of CD81. All tetraspanins have four transmembrane segments, a small extracellular loop (SEL) and a large extracellular loop (LEL). The tetraspanin LEL has a conserved domain (containing three helical segments) and a variable domain (VD; with two helical segments) which comprises the δ‐loop. The cartoon shows CD81 with the backbone of its LEL illustrated with the programme VMD (visual molecular dynamics) using the protein data bank (PDB) file 1G8Q 17; the δ‐loop is coloured in red. In the constructs CD9_Chim_ and CD151_Chim_, the δ‐loops of CD9 and CD151 were exchanged by the δ‐loop of CD81, respectively.

Comparing CD81‐RFP to either CD81‐GFP or CD81‐Δδ‐GFP (here a small 11 aa segment from the LEL was removed) we found that overlap is lost, suggesting targeting of CD81 into its clusters requires this region 15. However, successful targeting can have several reasons, for example, we cannot differentiate between a stabilizing effect or a preceding interaction mediating the specificity of the interaction. For clarification, we tested whether a ‘wrong’ tetraspanin could be targeted to CD81 clusters solely by carrying the CD81s’ δ‐loop. Using the tetraspanins CD9 and CD151, chimeras were generated replacing their δ‐loops by the one of CD81 (Fig. 1), in the following termed CD9_Chim_ and CD151_Chim_. We first compared CD81/CD9 to CD81/CD9_Chim_ asking whether we could increase the low overlap between CD81 and CD9 by using the CD9_Chim_ construct.

**Figure 2:**
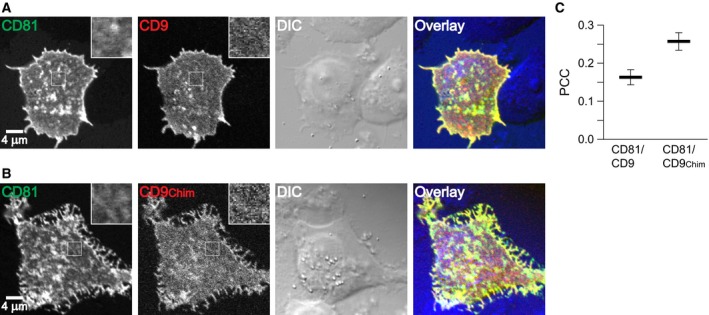
Analysis of intact cells shows that the δ‐loop of CD81 targets CD9 into CD81 clusters. Paraformaldehyde fixed Jurkat T cells cotransfected pairwise with (A) CD81‐GFP and CD9‐RFP or (B) CD81‐GFP and CD9_Chim_‐RFP. From left to right, overviews from the GFP‐ and RFP‐channels (insets show magnified views from the boxed regions), DIC recording and overlays. (C) The Pearson correlation coefficient (PCC) between the boxed regions as illustrated was calculated, yielding a quantitative value for the similarity between the green and red signal distribution (values can range from −1 to +1, indicating the pictures’ negative and identical pictures). Values are given as means ± SEM (*n* = 3; including the analysis of in total 127 cells; three independent experiments for each conditions, averaging 20–25 cells for each value).

**Figure 3:**
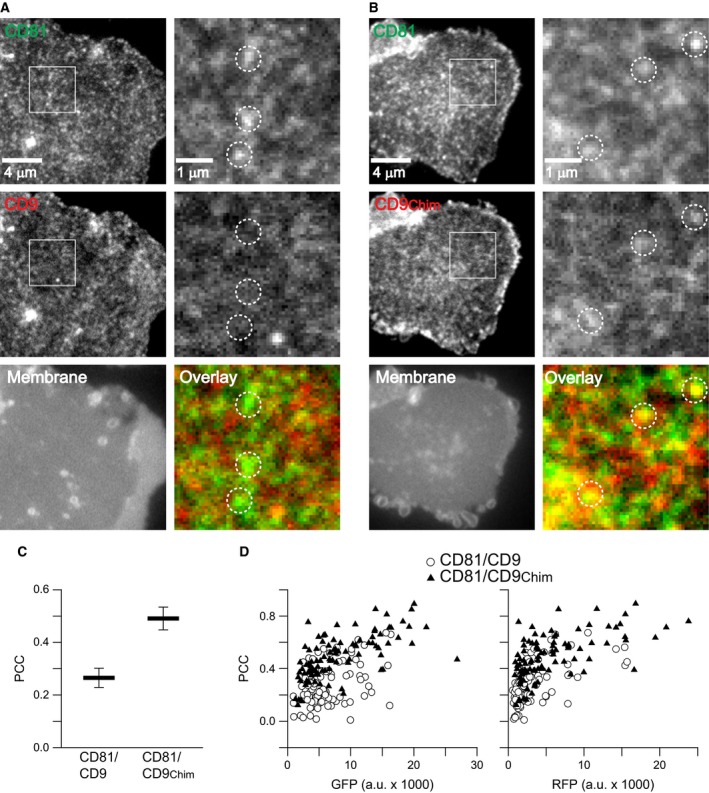
CD81 δ‐loop‐mediated targeting of CD9 into CD81 clusters analysed on membrane sheets. Membrane sheets from Jurkat T cells cotransfected pairwise with (A) CD81‐GFP and CD9‐RFP or (B) CD81‐GFP and CD9_Chim_‐RFP. Left, overviews from the GFP‐ and RFP‐channels, and a recording showing a general membrane stain by the dye TMA‐DPH. Right panels show magnified views from the GFP and RFP recordings and overlays. Dotted circles indicate identical pixel locations. Calculating the PCC, the similarity between green and red signals is quantified in (C). Values are given as means ± SEM (*n* = 4). (D) Plotting from the 192 membrane sheets collected from the four independent experiments in (C) their GFP (left graph) and RFP fluorescence (right graph) against their PCCs. Circles and triangles indicate membrane sheets from the conditions CD81/CD9 and CD81/CD9_Chim_, respectively.

We performed the experiment both in paraformaldehyde fixed cells (Fig. 2) and membrane sheets (Fig. 3). As previously observed 15 the PCC for CD81/CD9 was low both in cells (Fig. 2) and membrane sheets (Fig. 3), but when using CD9_Chim_ it increases by ≈ 0.1 in cells and more than 0.2 in membrane sheets. The PCCs in cells are lower due to the lower signal‐to‐noise ratio imaging in confocal microscopy that can be appreciated when comparing the images. The confocal GFP‐channel resembles a noisy and poorly resolved cluster pattern which is better detected with the CCD camera on membrane sheets (compare CD81‐GFP signal shown in insets from Fig. 2 with the magnified views of CD81‐GFP in Fig. 3). In case of the less potent RFP fluorophore, in the confocal microscope the signal does not even display discrete clusters, but only noisy areas with increased or decreased fluorescence intensity (see insets in the RFP channel in Fig. 2). Therefore, on intact cells it is not possible to obtain high PCC values by confocal microscopy. Nevertheless, the experiment shows that the improved targeting of CD9_Chim_ into CD81 clusters is not a phenomenon associated with membrane sheets.

The membrane sheet signal intensities vary with the expression levels of the GFP and the RFP constructs and these expression levels also influence the signal‐to‐noise ratio. To exclude that the lower PCC for CD81/CD9 is due to the imaging of dimmer membrane sheets, we compared the expression levels of CD81‐GFP/CD9‐RFP and CD81‐GFP/CD9_Chim_‐RFP from all membrane sheets included into the analysis. As shown in Fig. 3D, the expression levels were in the same range. Independent from the type of fluorescent label, the PCCs tended to be higher for the pair CD81‐GFP/CD9_Chim_‐RFP. Hence, the differences in the PCCs are not due to differences in expression levels.

**Figure 4:**
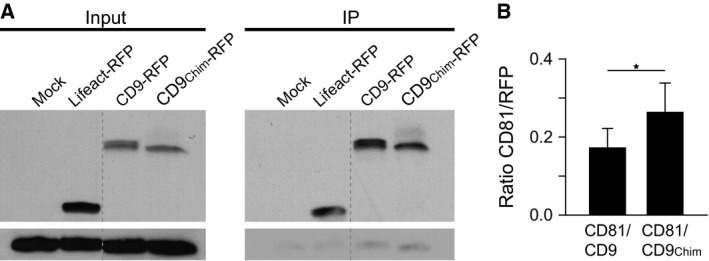
CD9 with CD81s’ δ‐loop coprecipitates endogenous CD81 more efficiently than CD9. Immunoprecipitation of endogenous CD81 by overexpressed RFP‐labelled CD9 or CD9_Chim_ using beads coated with an antibody directed against RFP. (A) Representative western blots. For the left and right panels parts from the same western blot membrane are shown, respectively. Left; input, showing as controls mock and Lifeact‐RFP‐transfected cells. Right, immunoprecipitation (IP). Top, RFP‐signal; bottom, endogenous CD81. (B) Signal of immunoprecipitated RFP constructs and coimmunoprecipitated endogenous CD81 related to each other. Values are given as means ±SEM (*n* = 7; paired *t*‐test (Shapiro–Wilk): *P* = 0.04 (*)).

We also aimed for confirming the δ‐loop effect applying IP after mild solubilization with CHAPS, which preserves the interactions between different individual tetraspanins. Jurkat T cells, cells were lysed in 1% CHAPS and after a short centrifugation step the RFP tag was precipitated and the pulled down endogenous CD81 was quantified. Compared to CD9, CD9_Chim_ was more efficient in pulling down endogenous CD81 (Fig. 4), albeit the δ‐loop‐mediated increase in immunoprecipitation is not as convincing as the produced increase in microscopic overlap.

**Figure 5:**
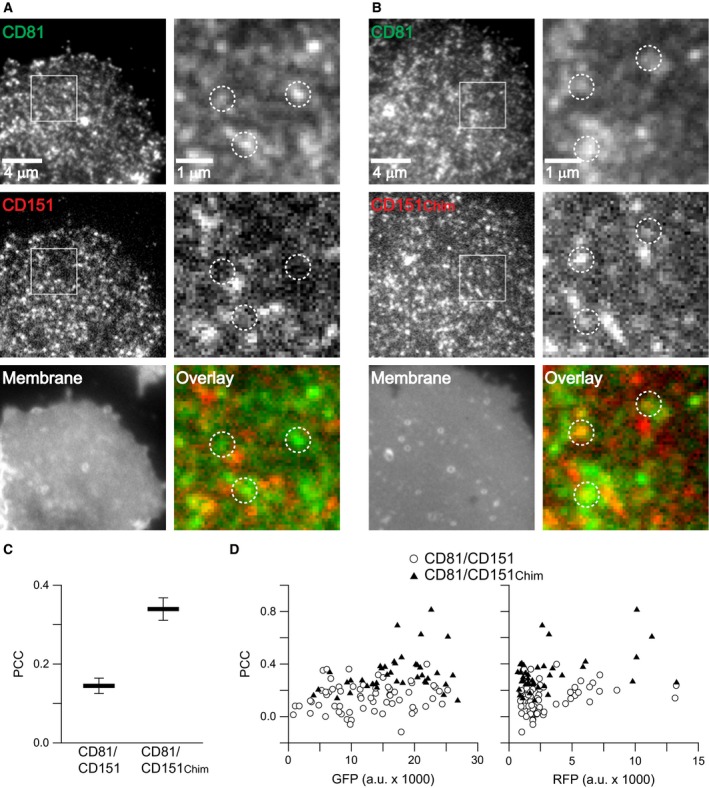
The δ‐loop of CD81 targets CD151 into CD81 clusters. Experiments as in Fig. 3, analysing membrane sheets from Jurkat T cells cotransfected with CD81‐GFP/CD151‐RFP (A) or CD81‐GFP/CD151_Chim_‐RFP (B). (C) Values are given as means ± SEM (*n* = 3). (D) From the 104 membrane sheets included in (C) GFP‐ (left graph) and RFP‐fluorescence (right graph) is plotted against PCC. Circles and triangles refer to membrane sheets from the conditions CD81/CD151 and CD81/CD151_Chim_, respectively.

When comparing the pairs CD81/CD151 and CD81/CD151_Chim_, a similarly strong gain in overlap was observed, although starting from an overall lower level (Fig. 5).

**Figure 6:**
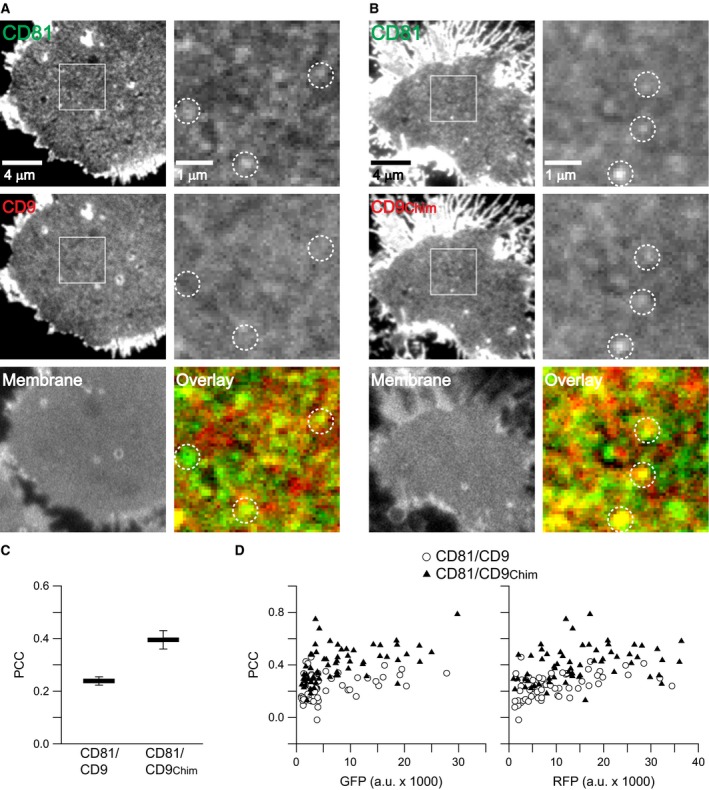
The δ‐loop‐mediated targeting of CD9 into CD81 clusters in HepG2 cells. Same experiment as illustrated in Fig. 3 with the exception that instead of Jurkat T cells HepG2 cells were used. (A) and (B) Fluorescence micrographs from membrane sheets. (C) Pearson correlation coefficients. Values are given as means ± SEM (*n* = 3; in total 121 membrane sheets were analysed). (D) Plotting from individual membrane sheets the PCC values versus their GFP‐ and RFP‐fluorescence intensity.

**Figure 7:**
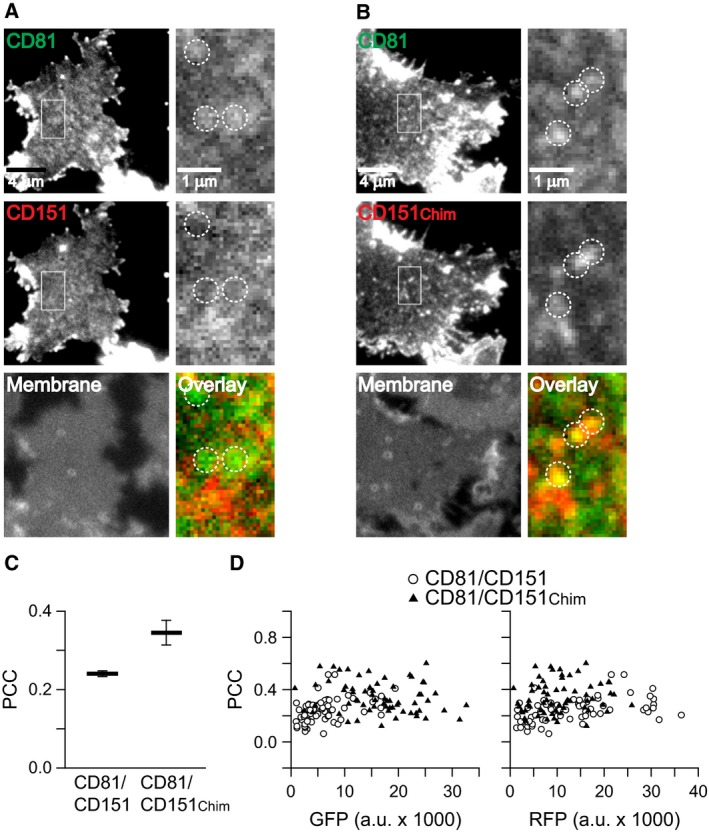
The δ‐loop‐mediated targeting of CD151 into CD81 clusters in HepG2 cells. Same experiment as illustrated in Fig. 5 using HepG2 instead of Jurkat T cells. (A) and (B) Fluorescence micrographs from membrane sheets. (C) Pearson correlation coefficients. Values are given as means ± SEM (*n* = 3; including the analysis of in total 131 membrane sheets). (D) The PCC values from individual membrane sheets plotted versus their GFP‐ and RFP‐fluorescence intensity.

We next turned to a different cellular system, namely HepG2 cells. These cells differ from Jurkat T cells as they have no endogenous CD81, and they are adherent cells, making the adhesion step prior to sonication redundant. We analysed the same construct pairs as in Jurkat T cells. As shown in Figs 6 and 7, the same basic finding is obtained, although the δ‐loop‐mediated gain in overlap was below 0.2, which is slightly less when compared to Jurkat T cells. This might be due to the stronger expression levels of the constructs in HepG2 cells leading to less distinct cluster patterns (compare Figs 3 and 5 to Figs 6 and 7).

In conclusion, analysis of microscopic overlap in two different cell systems shows that CD9 and CD151 do not overlap to a large extent with CD81, which is in line with earlier reports on the segregation of clusters formed by different tetraspanins 13, 15. Importantly, overlap increases strongly when CD9 and CD151 carry the δ‐loop of CD81.

The data show the δ‐loop plays a pivotal role in homophilic clustering and carries the information for targeting CD81 into CD81 clusters. As previously suggested by the cluster phase model, clusters are composed of transiently forming binary complexes (in this case homo‐dimers and/or CD81‐primary partners) that exchange molecules with each other 15. In this model, the specificity of the δ‐loop would allow only CD81 molecules to enter the cluster phase that likely also contains other, but not tetraspanin, molecules. However, the model would be also in line with a cluster composed exclusively of CD81.

It should be noted that the δ‐loop is not alone responsible for cluster building and maintenance, as de‐palmitoylation of tetraspanins leads to smaller clusters (from 140 to 97 nm; 12) and a small diminishment in CD81 cluster targeting efficiency 15.

The δ‐loop is located within the variable domain of the LEL, which has been suggested to be important for partner selection and initial contact establishment 18, 19, 26, 27. In line with the tetraspanin‐partner pair model 1, a CD81‐partner complex would be connected via a specific δ‐loop‐mediated dimerization step to another CD81‐partner complex, of identical or different composition. Alternatively, an already formed CD81 dimer may bind to primary partners. As has been pointed out, by this mechanism, tetraspanins, or more precisely functional dimers 14, have a specific role in partner selection and a nonspecific role in regulating the stoichiomertry of the interactions that define the tetraspanin web. This model is compatible with the cluster phase model outlined above, with the only exception that in the cluster phase model at any moment only binary complexes are present, that in the next moment may dissociate in order to form new combinations of binary complexes.

## Author contributions

YH and TL conceived the project and designed the experiments. YH performed and analysed the experiments. YH and TL wrote the article.
